# Eco-friendly monitoring of triclosan as an emerging antimicrobial environmental contaminant utilizing electrochemical sensors modified with CNTs nanocomposite transducer layer

**DOI:** 10.1186/s13065-023-01092-0

**Published:** 2023-11-28

**Authors:** Nardine Safwat, Amr M. Mahmoud, Maha F. Abdel-Ghany, Miriam F. Ayad

**Affiliations:** 1 Pharmaceutical Analytical Chemistry Department, Faculty of Pharmacy, Ain Shams University, Abbassia, 11566 Cairo Egypt; 2 Pharmaceutical Analytical Chemistry Department, Faculty of Pharmacy, Cairo University, Kasr-El Aini Street, Cairo, 11562 Egypt

**Keywords:** Antimicrobial resistance, Coronavirus disease-19, Emerging contaminants, Green analysis, Solid contact ISEs, Triclosan

## Abstract

Environmental appearance of antimicrobials due to frequent use of personal care products as recommended by WHO can cause serious flare-up of antimicrobial resistance. In this work, three eco-friendly microfabricated copper solid-state sensors were developed for measuring triclosan in water. Multi-walled carbon nanotubes were incorporated in sensor 2 and 3 as hydrophobic conductive inner layer. Meanwhile, β-cyclodextrin was incorporated in sensor 3 as an ionophore for selective binding of TCS in presence of interfering compounds. The obtained linear responses of sensors 1, 2 and 3 were (1 × 10^− 8^–1 × 10^− 3^ M), (1 × 10^− 9^–1 × 10^− 3^ M) and (1 × 10^− 10^– 1 × 10^− 3^ M), respectively. Limit of detection was 9.87 × 10^− 9^ M, 9.62 × 10^− 10^ M, and 9.94 × 10^− 11^ M, respectively. The miniaturized sensors were utilized for monitoring of triclosan in water samples.

**Supplementary Information:**

The online version contains supplementary material available at 10.1186/s13065-023-01092-0.

## Introduction

The outbreak caused by the emergence of COVID-19 disease was announced by the WHO as being pandemic in March 2020. Owing to the absence of vaccines or drugs approved for the treatment of COVID-19 disease to face the global dramatic increase in the infection rates, the WHO has recommended to follow healthy lifestyles and safety measures in order to prevent the global spread of COVID-19. Excessive use of hand sanitizers, soaps and detergents has raised a great concern to the emergence of antimicrobial resistance which pose a serious threat to public health and global economy. Triclosan; an antimicrobial agent, has been reported as an emerging contaminant in the environment and that issue has escalated and has become disastrous during the pandemic outbreak of COVID-19 [1–3].

The emergence of drug residues, cosmetic products, steroids, surfactants, fragrances, and plasticizers in the environment have been growing in the past decades [4, 5]. These emerging environmental contaminants are any uncommon man-made or natural chemicals or any microorganisms that have the ability to reach the environment and may pose uncommon harmful ecological and human health effects [6]. The scientific community is paying a great attention to this abnormal outstanding phenomenon of environmental pollution. Consequently, numerous environmental and toxicological studies were carried out universally to face this challengeable problem for the human safety and environmental protection [7].

Triclosan (TCS) is an antimicrobial compound with a broad-spectrum activity employed in various cosmetic and personal hygiene products such as toothpastes, deodorants, skin cosmetics and antibacterial soaps. Moreover, it is found in toys, textiles, plastics, and kitchen utensils [8]. It is considered as one of the most remarkably detected emerging contaminant in the environment [9]. It was reported that TCS and its degradation byproducts are highly toxic producing cytotoxic, genotoxic, and endocrine disruptor effects [8]. TCS was found to pose toxic effects in humans as well as aquatic species [10–13]. Furthermore, emergence of resistant bacterial strains was observed [14].

Owing to the higher toxicity of TCS, its use in Minnesota was banned by 2014, and it was applied in 2017. In Europe, TCS usage was limited in cosmetics [15]. Chlorophenols, which are metabolites of TCS, were listed to the priority toxic pollutants published by the United States Environmental Protection Agency (US- EPA) due to their higher toxicity and bioaccumulation [8].

A literature survey has shown that various techniques for TCS determination have been applied in different matrices including separation techniques such as HPLC and GC coupled with tandem mass spectrometric or ultraviolet detectors [16–21], some spectroscopic [22–25] and electrochemical reports have been published [26–28].

Electrochemical sensors are considered as a green method of analysis. Electrochemical sensors are characterized by their capability of inline monitoring of the analytes, cost-effective, minimal production of wastes and highly sensitive and selective. Potentiometric sensors (such as ion-selective electrode) are appealing for inline and continuous detection of emerging environmental contaminants. Solid contact ISEs offers several advantages over liquid state ISEs counterparts of being stable, rigid, simple, without inner filling solution and easy to miniaturize [29]. However, the existence of an aqueous layer between the solid-contact electrode and the polymeric ion selective membrane (ISM) leads to the occurrence of a potential drift which is considered as one of the principal drawbacks of solid-contact sensors. The formed water layer deteriorates both the stability and sensitivity of the sensor. Minimization of the negative impacts of the aqueous layer can be performed by incorporating a hydrophobic conductive interlayer at the interface of ISM and the solid contact. Multi-walled carbon nanotubes (MW-CNTs) have been employed as a lipophilic ion-to-electron transducer layer at the interface of ion-selective membrane/solid-contact [30]. Furthermore, miniaturization and microfabrication have drawn great attention especially in the field of applying green chemistry. Microfabricated sensors are small, rapid, easy to use and environmentally friendly. Copper is not commonly used as electrode substrate, but there has been recent interest to utilize it as substrate due to its advantages such as cheapness compared to platinum and gold, applications in microfabrication process and printed circuit board design, and wide applications. It is worth mentioning that the literature contains few potentiometric sensors designed for TCS detection, such as liquid contact ISE impregnated with a molecularly imprinted polymer (MIP) for selective TCS determination, this proposed sensor has LOD equal 1.9 × 10^− 9^ M, in spite of the fact that reported LOD is low, the MIP preparation methodology is laborious [31].

Nanoparticles have been attractive to most of the researchers owing to their incomparable characteristics and broad real-world applications. The choice and optimization of the components of a developed sensor are prerequisite for obtaining a sensor with an excellent performance [32]. An example of nanoparticles that have gained a great interest in different analytical techniques is carbon nanotubes (CNTs). CNTs are characterized by their unique structure with significant thermal and electrical properties [33]. CNTs nanocomposites have been deposited on the surface of SC-ISEs as they can minimize the signal drift which is one of the main drawbacks of SC-ISEs [34]. CNTs have been employed in ISEs as an ion to electron transducing coat and since they are lipophilic, the aqueous layer that tend to be formed between the electrode and the membrane was no longer present and therefore the long-term signal stability of the electrochemical sensor was strongly enhanced. CNTs are stable under atmospheric O_2_ and CO_2_ since they are nonreactive and do not experience any oxidation or reduction reactions. Furthermore, CNTS are not light sensitive. All the above disclose why CNTs are being superior to other polymers that have been utilized as transducers [35].

From this point, it is necessary to design an eco-friendly, accessible, sensitive, and rapid potentiometric sensor using three micro-fabricated solid contact potentiometric sensors for measuring and monitoring the emerging contaminant TCS in the environmental water samples. The sensor was developed and optimized on two steps: first, the MW-CNTs nanocomposite layer was incorporated for sensitivity and potential stability enhancement and the sensor performance was compared to a bare Cu solid state ISE. Second, we exploited the strong complexation between TCS and β -cyclodextrin (β-CD) for enhancing both the sensitivity and selectivity of the proposed sensor by incorporating β-CD in the PVC polymeric membrane [36, 37]. The proposed sensors have been validated in accordance with the IUPAC recommendations and then, exploited for TCS measurement in real environmental samples. The sensors’ response was statistically compared with reported work to evaluate the validity and applicability of the microfabricated SC-ISEs.

## Experimental

### Materials

Triclosan (TCS), Multi-Walled-Carbon nanotubes (MW-CNT) and β-cyclodextrin (purchased from Sigma-Aldrich (Germany). Tetrahydrofuran (THF), 1-Nitro-2-octyloxybenzene (*o*-NPOE), tetra-dodecylammonium chloride (TDAC), and high molecular weight polyvinyl chloride (PVC) were obtained from Fluka. Deionized water. Other chemicals are mentioned in the supplementary materials.

### Instrumentation

- Ag/AgCl double junction – type external reference electrode (Thermo Scientific Orion 900,200, (MA, USA); 3.0 M KCl saturated with AgCl as an inner filling solution and 10% KNO_3_ as a bridge electrolyte) and Jenway digital ion analyzer (model 3330, Essex, UK) were used for potentiometric measurements.
- For pH adjustments, a Jenway pH glass electrode no. 924,005- BO3- Q11C (Essex, UK) was used.
- For temperature adjustments, a Bandelin Sonorex magnetic stirrer and heater, model Rx510S (Budapest, Hungaria) was used.

### Preparation of TCS stock standard solution

Preparation of 1 mM stock solution of TCS was carried out by dissolving 28.0 mg of TCS in 3 mL of NaOH and then the complete the volume to 100 mL using 0.1 M Na_2_CO_3_ / NaHCO_3_ buffer (pH 10.0) in volumetric flask. The TCS stock solution was stored at 4 °C in the fridge.

### TCS working solutions

The working solutions (1.0 × 10^− 4^ − 1.0 × 10^− 10^ M) were prepared from the TCS stock solution (1 mM) by dilution using 0.1 M Na_2_CO_3_ / NaHCO_3_ buffer.

### Water samples collection

Water samples have been collected in amber glass bottles that has been pre-rinsed with ultra-pure water, the bottles were completely filled without any headspace remaining. Three water sources were selected, the first source was the tap drinking water sample collected from domestic water supply. The second source of water sample is water from drinking water plant before treatment, that plant receives water supply from pipes 100 Km away from the Nile River, while the third source is water sample collected from drinking water plant just after water treatment and before distribution to other areas.

All samples have been refrigerated (at 4 °C) during transfer to the lab. Then, samples were vacuum filtered through 0.45 μm membrane filter and stored at 4 °C without any preservative and analyzed within two days to prevent microbial contamination.

### Procedures

#### Microfabrication of the copper electrodes

**Fig. 1:**
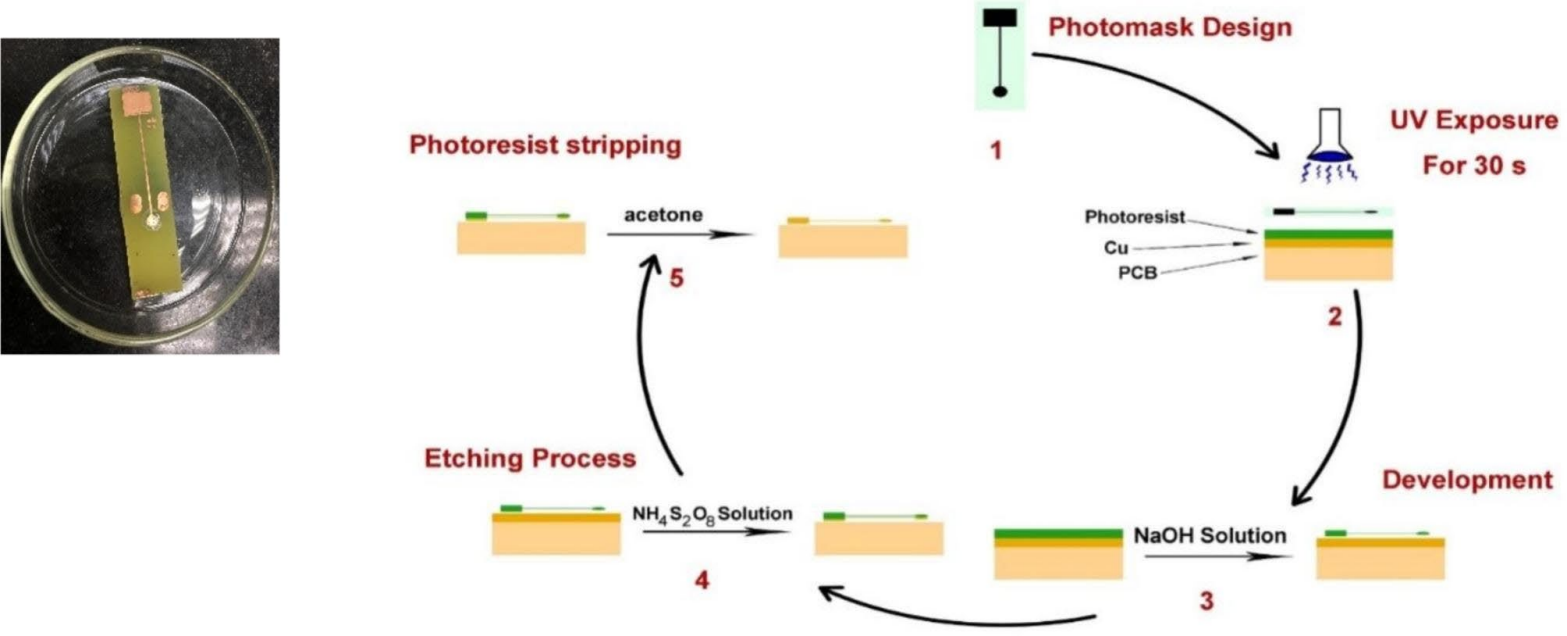
Schematic diagram of the microfabrication steps of the proposed sensors with an insert showing the real photo of the microfabricated sensor

The design of photomask was carried out using CAD software with specified sensor dimensions then printed using a laser printer on a transparent sheet. Pre-sensitized photoresist coated printed circuit boards (PCB) was covered with the photomask and exposure to ultraviolet light (365 nm) for 30 s was utilized to transfer the designed sensor pattern. The photomask blocks the UV irradiation on the defined dimensions, while exposes the uncovered positive photoresist. The photoresist development process was performed by agitation the PCB in NaOH solution (0.25 M) for 2 min to take off the UV irradiated photoresist. Then, exposed copper was etched using wet etching process by soaking PCB in ammonium persulfate solution (1.1 M) at 40 °C. Finally, the acetone was used to strip the remaining photoresist and expose the microfabricated copper electrode. Washing of the microfabricated Cu electrodes were done thoroughly with isopropyl alcohol and double-distilled water before use. The microfabrication steps are summarized as shown in (Fig. 1).

#### CNT-nanocomposite preparation and ion selective membrane composition

First, MW-CNT nanocomposite was prepared as reported in the literature, briefly, mixing 0.15 mg of MW-CNT with 5 mg *o*-NPOE (5 μL) in 1 mL THF and then sonicated for 20 min.

For both sensor 1 and 2, ISM was prepared by mixing *o*-NPOE (66.60%, 399.6 mg) with PVC (33.24%, 199.44 mg) and tetradodecylammonium chloride (0.16%, 0.96 mg) in a test tube. For sensor 3, it is prepared by mixing *o*-NPOE (66.60%, 399.6 mg) with PVC (32.19%, 199.44 mg), tetradodecylammonium chloride (0.16%, 0.96 mg), and the ionophore β-CD (1.05%, 6.35 mg) in a test tube, the ion exchanger to ionophore ratio was chosen to be 1:2 in order to optimize the ionophore-doped sensor response as reported in our previous work [38].

The assembly of the solid-contact potentiometric sensors was performed by direct drop-casting 10 μL of the ISM on Cu electrodes for sensor 1 and let the ISM to dry for 24 h. While for sensors 2 and 3, a layer of CNT-nanocomposite was drop-casted (10 μL) on Cu electrodes first and dried in air for 24 h, and then a 10 μL of the corresponding ISM was drop-casted on modified Cu electrode.

#### Calibration

Calibration curves for TCS have been constructed by measuring the potential difference (electromotive force, *emf*) between prepared the TCS serial dilutions against double junction reference electrode. The interfering anions calibration curves have been recorded by serial dilution of the interfering ion solution using deionized water. Selectivity coefficients have been computed using the separate solution method [39]. Limit of detection (LOD) has been calculated in accordance with the IUPAC recommendations [40].

#### Analysis of water samples using the microfabricated sensors

A volume of 5 mL of 1 × 10^− 6^, 1 × 10^− 5^, 1 × 10^− 4^ M standard solutions have been transferred separately to 50 mL volumetric flasks then the volume was completed by the drinking tap water sample (whose pH was 10.0) to yield final concentrations 1 × 10^− 7^, 1 × 10^− 6^, and 1 × 10^− 5^ M, respectively. The three sensors were utilized to measure *emf* values of the prepared samples. Applying the calculated regression equations, the concentrations have been computed utilizing the obtained *emf* results. The concentration results are presented as recovery ± standard deviation.

#### Investigation of the electrochemical figures of merit of fabricated sensors

According to IUPAC recommendations [41], the proposed microfabricated sensors’ figures of merit such as slope, response-time, and other parameters have been evaluated.

#### Study of pH effect on sensor’s performance

Studying the pH effect on the microfabricated sensors’ electrochemical signal response was performed at two different concentrations (1 × 10^− 4^ and 1 × 10^− 5^ M) at the pH range from 1.0 to 12.0. For each concentration, the *emf* was measured as a function of pH.

#### Effect of temperature

Studying the thermal effect on the designed microfabricated sensors’ response was carried out. The potential response of working solutions within different concentration ranges (1 × 10^− 8^ – 1 × 10^− 3^ M) for sensor 1, (1 × 10^− 9^ – 1 × 10^− 3^ M) for sensor 2 and (1 × 10^− 10^ – 1 × 10^− 3^ M) for sensor 3 was measured at these temperature values (25, 30, 35, 40, and 45 °C). For each temperature, sensor calibration has been carried out.

#### Potentiometric aqueous layer test

The solid-state ISEs’ long-term stability is highly impacted by the presence of a water layer underneath the ISM [42]. The aqueous layer formation can be assessed by measuring the potential signal drift of the sensor upon changing from the ion of interest (1 × 10^− 6^ M) to an interfering ion solution with a high concentration (1 × 10^− 2^ M of naproxen (NPR)) and then immersed into the primary ion of interest. If there was a water layer undesirable potential signal drifts will occur.

## Results and discussion

The proposed microfabricated solid-contact ISEs can determine TCS in drinking tap water samples with high sensitivity, selectivity, and lower detection limits.

### Microfabrication of the miniaturized solid-contact sensors

**Fig. 2:**
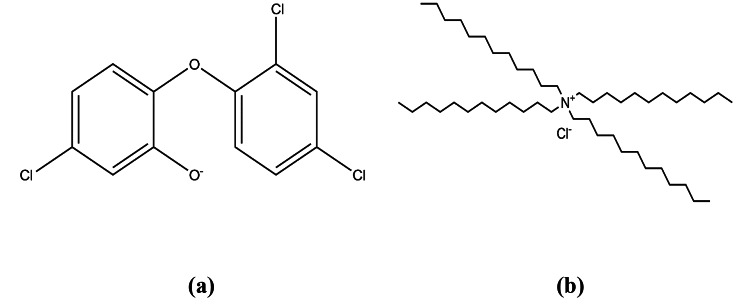
Chemical structure of (**a**) TCS showing its anionic site and (**b**) Tetra-dodecyl ammonium chloride

The proposed study is based on the interaction between the anionic site present in the TCS chemical structure and the cationic exchanger tetra-dodecyl ammonium chloride as shown in Fig. 2. Their interaction was proved to be optimum and stable, by the observed Nernstian responses for the three microfabricated sensors.

The rationale behind the design of sensor 2 is that the addition of MW-CNTS might offer several advantages versus the control sensor (sensor 1). Since SC-ISEs response generally negatively influenced by the potential signal drift, plausibly due to the existence of an aqueous film beneath the membrane which may affect SC-ISEs long-term signal stability. Incorporation of hydrophobic nanomaterials that possess both electronic and ionic conductivity might act as ion-to-electron transducers between the ion selective membrane and the electronic conductor. These nanomaterials have greatly enhanced the long-term potential signal, sensitivity, and improved detection limits of the solid-state sensors [42–44]. MW-CNTs were a good candidate to act as a redox-buffering underlayer improving the long-term potential stability, the selectivity, sensitivity, and detection limits of the proposed sensor.

Supramolecular macrocycles embedded within electrochemical sensors have been reported to enhance both sensitivity and selectivity via formation of inclusion complexes between the supramolecular host and the analyte guest [45]. For the proposed sensor 3, β-CD was impregnated into the ISM as an ionophore to increase the selectivity of the membrane and hence enhance the sensitivity of the electrode. The choice of β-CD as ionophore is based on the reported great affinity to β-CD towards TCS creating an inclusion complex in a 1: 1 ratio [36, 46].

### Sensors’ calibration and response time

**Fig. 3:**
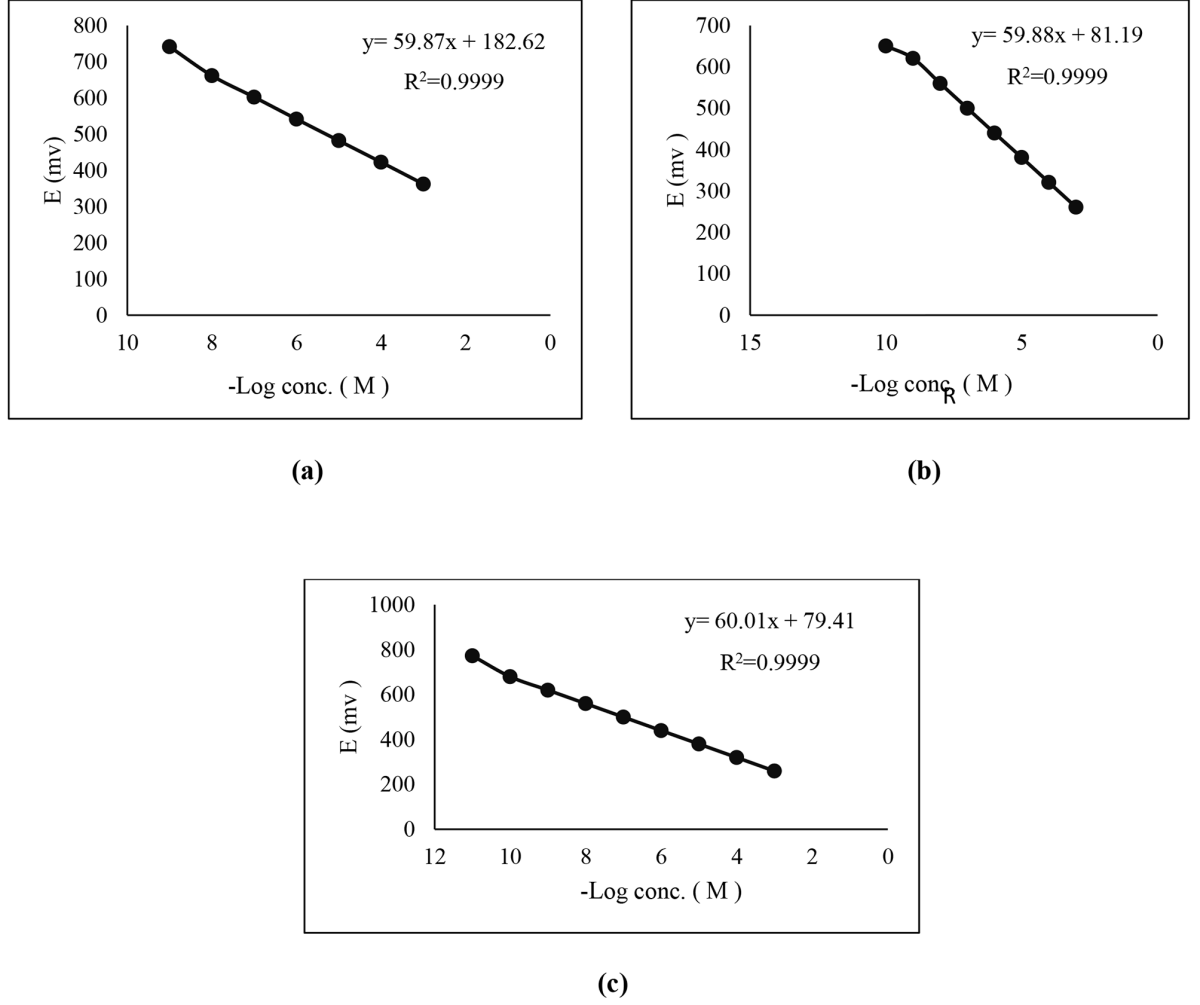
Profile of the potential in mV versus -Log concentration of TCS using the proposed microfabricated (**a**) sensor 1 (**b**) sensor 2 (**c**) sensor3

The electrochemical characteristics of microfabricated sensors has been evaluated in accordance with the guidance of the IUPAC recommendations [47] and are represented in Table 1. Calibration curves of the proposed microfabricated sensors were plotted as shown in Fig. 3, which represents linear responses and almost ideal behavior of the monovalent anionic Nernstian slopes recorded in Table 1. The LOD was computed from the intersect of the two linear extrapolated parts of the calibration curve. The response time is the time required for the sensor to reach equilibrium potential value (within ± 1 mV of the final signal) after a 10-fold increment in the analyte concentration. The sensors response time was estimated.

**Table 1:** General characteristics of the three microfabricated proposed sensors

Parameter	Sensor 1	Sensor 2	Sensor 3
Slope (mV/ decade) ± SD ^*^	59.87 ± 0.26	59.88 ± 0.19	60.01 ± 0.11
Intercept (mV)	182.62	81.19	79.41
LOD (M) ^**^	9.87 × 10^− 9^	9.62 × 10^− 10^	9.94 × 10^− 11^
Response time (sec.)	12	10	10
Working pH range	7.0–11.0	7.0–11.0	7.0–11.0
Concentration linear range (M)	1 × 10^− 8^ – 1 × 10^− 3^	1 × 10^− 9^ – 1 × 10^− 3^	1 × 10^− 10^ – 1 × 10^− 3^
Stability (days)	45	60	60
Average recovery (%) ± SD	99.98 ± 1.34	100.01 ± 1.35	99.99 ± 0.27
Correlation coefficient	0.9999	0.9999	0.9999
Repeatability (% RSD)	1.21	0.81	0.67
Intermediate precision (% RSD)	1.51	0.97	0.95

^*^Average of three determinations

^**^Limit of detection measured by intersection of the two linear extrapolated arms of Fig. (2)

### Effect of pH on sensors response

**Fig. 4:**
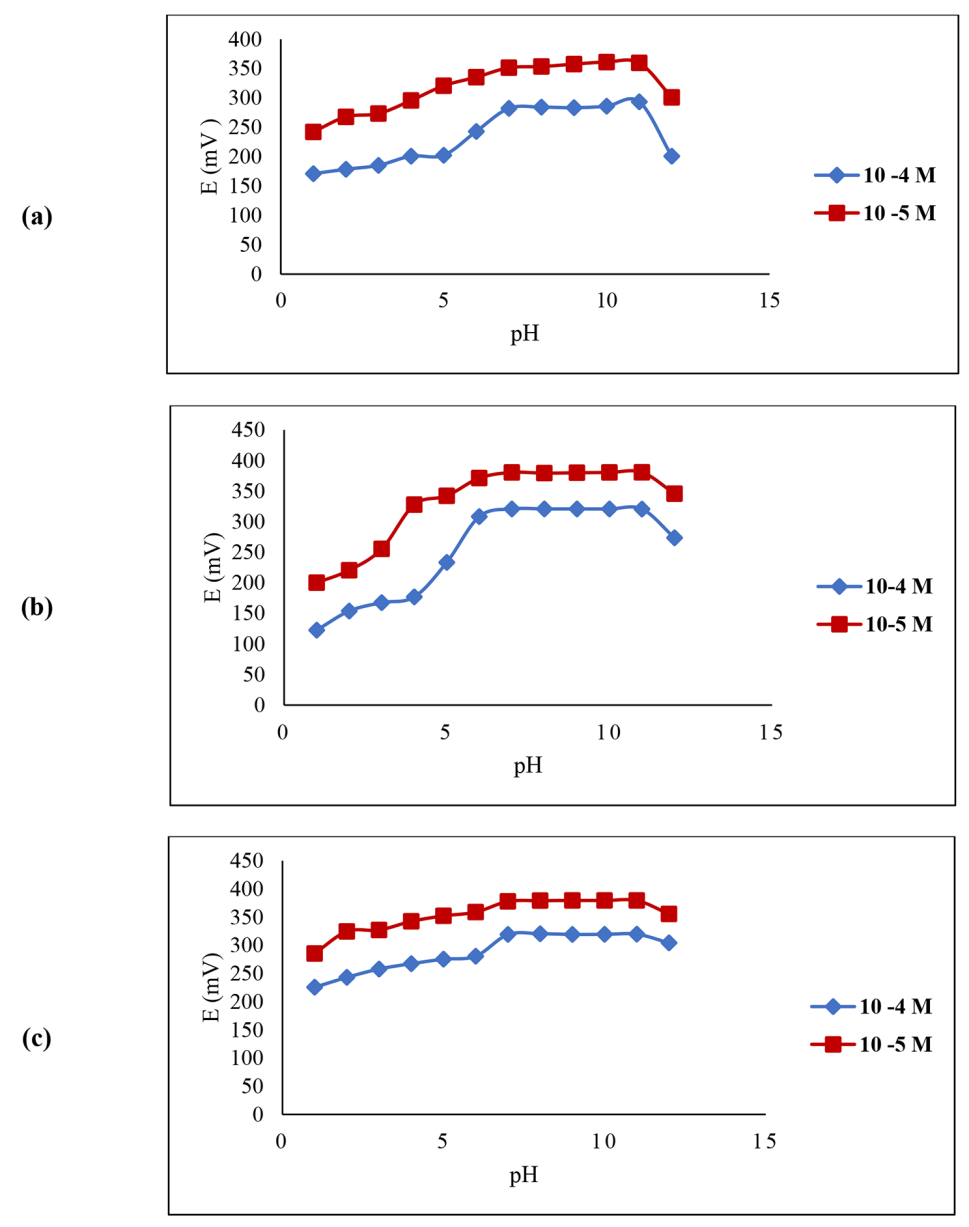
Effect of pH on the response of the proposed microfabricated (a) sensor 1 (b) sensor 2 (c) sensor3 for TCS

The pH influence on the potential signal of the three microfabricated sensors was investigated on two concentration values (1 × 10^− 4^ and 1 × 10^− 5^ M) at pH range (1.0–12.0), as shown in Fig. 4. It was concluded that pH range (7.0–11.0) is the optimal pH working range for all microfabricated sensors owing to the constant measured potential. Hydronium and hydroxyl ions may interfere at both extremes of pH values. Moreover, TCS is a phenolic derivative, once introduced into acidic pH, it becomes unionized, and hence precipitation takes place.

### Sensors response at various temperatures

**Fig. 5:**
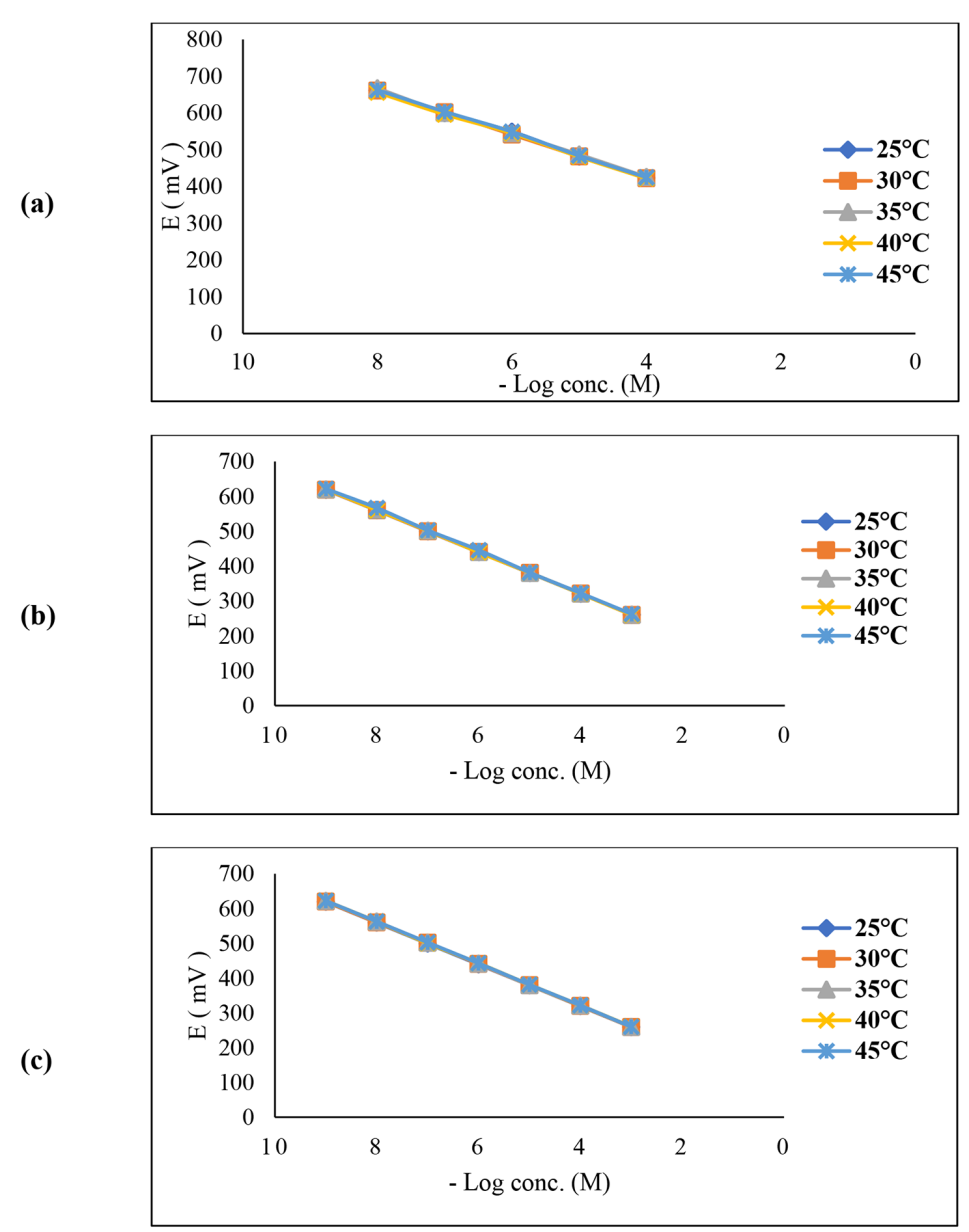
Effect of temperature on the response of the proposed microfabricated (**a**) sensor 1 (**b**) sensor 2 (**c**) sensor3 for TCS

Upon studying the thermal effect on sensors response, all proposed sensors exhibited little increase of *emf* potential response by elevation of the temperature from 25 to 45 °C. It’s worth noting that the obtained calibration graphs were parallel at the studied temperatures as represented in Fig. 5.

### Effect of foreign compounds on sensor selectivity

The influence of different interfering ions upon the application of the microfabricated sensors was evaluated by SSM [41]. Results are summarized in Table 2, these results show that the sensors selectivity coefficients in the presence of some inorganic anions (Cl^−^, NO_3_^−^, Br^−^, SO_4_^2−^, C_2_O_4_^2−^, C_2_H_3_CO_2_^−^, PO_4_^3−^) and an emergent pollutant that might coexist (i.e., Ketoprofen). The obtained results clearly indicate that the fabricated sensors exhibit high sensitivity towards the target contaminant. Moreover, sensor 3 possesses the highest selectivity in comparison to sensors (1 and 2), which can be attributed to the inclusion of β-CD as an ionophore.

**Table 2:** Potentiometric selectivity coefficients (Log K^**pot**^_**primary ion, Interferent**_**) for the three microfabricated proposed sensors**

Interferent^*^	Selectivity coefficient (Log K^pot^_primary ion, Interferent_) for sensor 1	Selectivity coefficient (Log K^pot^_primary ion, Interferent_) for sensor 2	Selectivity coefficient (Log K^pot^_primary ion, Interferent_) for sensor 3
Cl^−^	-3.334	-4.387	-4.511
NO_3_^−^	-4.139	-3.804	-4.359
Br^−^	-3.789	-4.913	-4.518
SO_4_^2−^	-5.393	-5.711	-4.722
C_2_O_4_^2−^	-5.328	-6.412	-4.451
C_2_H_3_CO_2_^−^	-4.631	-3.480	-4.314
PO_4_^3−^	-7.108	-6.135	-4.499
Ketoprofen	-4.385	-3.449	-3.763

^*^ Aqueous solutions of 1 × 10^− 4^ M were used

### Potentiometric aqueous layer test

**Fig. 6:**
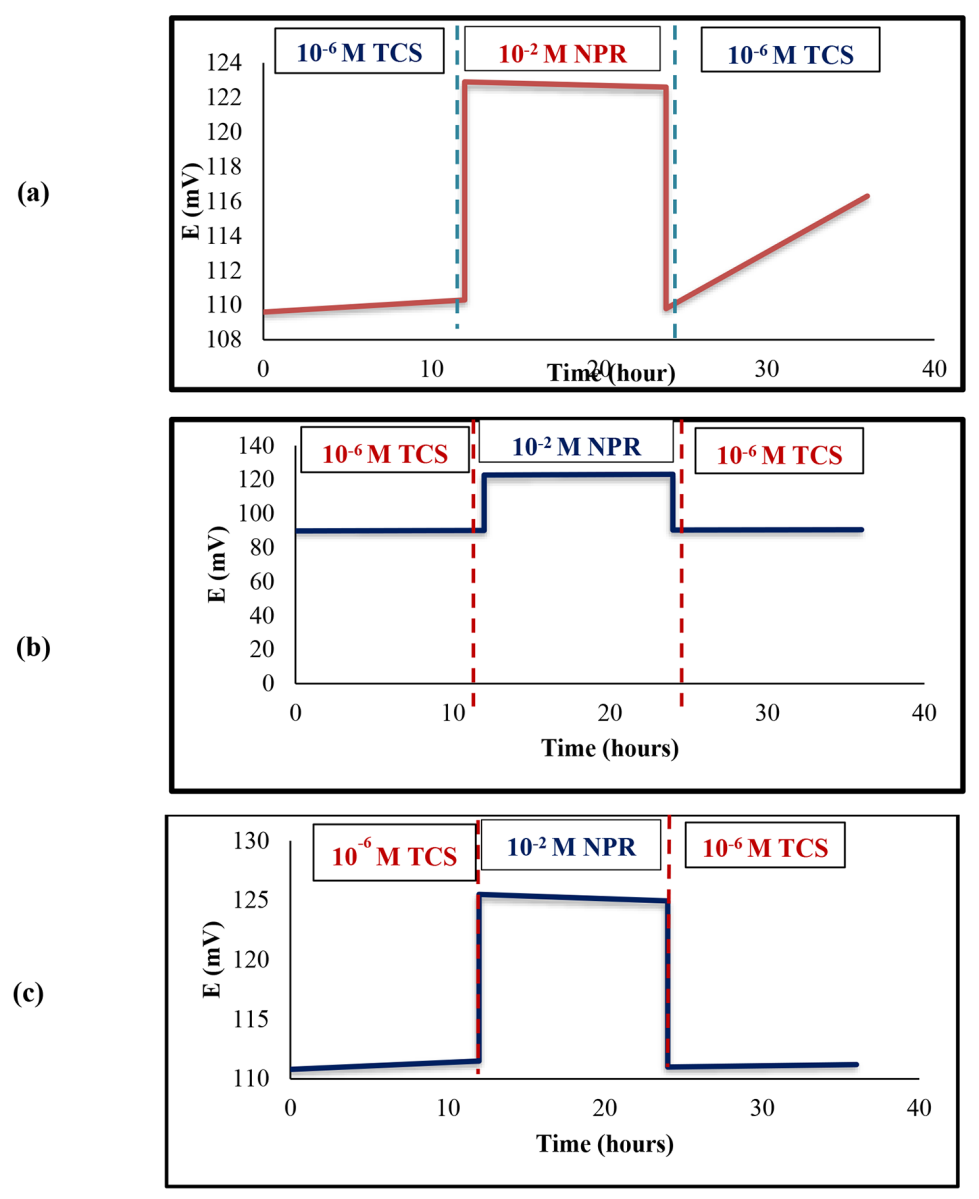
Aqueous layer test of the proposed microfabricated (**a**) sensor 1 (**b**) sensor 2 (**c**) sensor 3

There were not any noticeable potential drifts in the proposed sensors 2 and 3 potential response indicating that the incorporation of MW-CNT nanocomposite excluded the aqueous layers. However, due to the absence of MW-CNTs in the proposed sensor 1, an aqueous layer was formed leading to a highly significant potential drift affecting the long-term signal stability of the fabricated sensor as shown in (Fig. 6).

### Application to the spiked water samples

The proposed sensors can be employed to determine the target contaminant in water samples with acceptable recovery percentages with minimal interferences as shown in Table 3.

As sensor 3 is highly selective, sensitive, and accurate, it has been used for TCS determination in Nile River water samples and the treated water from the water treatment plant. TCS was found in Nile River water and treated water at concentrations 3.38 × 10^− 6^ M and 1.36 × 10^− 8^ M, respectively.

**Table 3:** Application of the three microfabricated proposed sensors on drinking water samples

TCS added conc. (M)	Sensor 1		Sensor 2		Sensor 3	
	TCS found conc. (M)	Recovery^*^ %	TCS found conc. (M)	Recovery^*^ %	TCS found conc. (M)	Recovery^*^ %
1 × 10^− 8^	1.00 × 10^− 8^	100.00	1.00 × 10^− 8^	100.00	9.82 × 10^− 9^	98.20
1 × 10^− 6^	1.01 × 10^− 6^	101.00	9.91 × 10^− 7^	99.10	1.00 × 10^− 6^	100.00
1 × 10^− 5^	1.01 × 10^− 5^	101.00	1.00 × 10^− 5^	100.00	9.94 × 10^− 6^	99.40
Mean ± SD	100.67 ± 0.58		99.70 ± 0.52		99.20 ± 0.92	

^*^ Average of three determinations

## Statistical analysis

The results of the three proposed microfabricated sensors and the reported method were statistically compared [48]. It was performed at 95% confidence level using the student’s t-test and F test as represented in Table 4. The results indicate that the difference was statistically insignificant between the fabricated and the reported method indicating good accuracy and precision.

**Table 4:** Statistical analysis of the results obtained by the proposed microfabricated sensors and the official method for the analysis of TCS

Statistical term	Official method ^**^	Proposed sensor 1	Proposed sensor 2	Proposed sensor 3
Mean	100.24	99.98	100.01	99.99
SD	0.483	1.348	1.353	0.274
Variance	0.23	1.28	1.08	0.04
n	5	5	5	5
t		1.12 (2.31) ^*^	1.62 (2.31) ^*^	1.14 (2.31) ^*^
F		5.49 (6.39) ^*^	4.64 (6.39) ^*^	5.35 (6.39) ^*^

^*^The theoretical values of t and F at *P* = 0.05

^**^High performance liquid chromatography determination of TCS [48] by using a C18, 5 μ (250 × 4.6 mm) column, a mobile phase of methanol: water (90: 10, v/v) at flow rate 1 mLmin^− 1^ and UV detection at λ = 280 nm using 20 μL as an injection volume

## Greenness assessment

Electroanalysis is a promising technique of green analysis since it is a direct method where no sample collection and pretreatment steps are needed. Miniaturization of the developed sensors is eco-friendly offering the advantage of real-time measurements, the use of small sample volume and reduction of the amount of waste. The microfabrication has eliminated the use of an inner filling solution and offering the advantage of being green, simple, low cost and sensitive. Environmental monitoring is in deep need for green analytical methods development instead of the conventional hazardous methods [29, 49]. Evaluation of proposed methods using specialized greenness assessment tools is highly recommended. The National Environmental Methods Index (NEMI) is oldest greenness assessment tool [50]. Later, Analytical Eco-scale was developed. The latter was based on computing a method score by assigning penalty points to various factors in the analytical procedure to be subtracted from a 100 base [51]. Recently Green analytical procedure Index (GAPI) was developed for the greenness assessment [52]. Analytical eco-scale and GAPI tools were adopted for the greenness assessment of the proposed method.

### Analytical eco-scale

Various factors included in the developed method were given penalty points to be subtracted from a 100 base. Excellent green analysis will be higher than 75 score, acceptable green analysis is higher than 50 and inadequate green analysis is less than 50. Penalty points are calculated based on both the type and amount of the reagent, the energy requirement of various electrical instruments, the treatment of the analytical waste and the occupational hazard [53]. The developed method eco-scale values were computed and were found to be 95 (Detailed information are provided in Table 5) and based on this assessment the proposed sensors have proved to be an excellent green analytical platform.

**Table 5:** The penalty points for the proposed potentiometric method using the Analytical Eco-scale method of assessment

Hazard	Penalty points for the proposed potentiometric sensors
• **Reagents**:	
Na_2_CO_3_ / NaHCO_3_ buffer, pH 10	2
Water	0
• **Instruments**:	
Energy and occupational hazard	0
Waste	3
Total penalty points	5
Analytical eco-scale for the proposed methods	**95**

### Green analytical procedure index

**Fig. 7:**
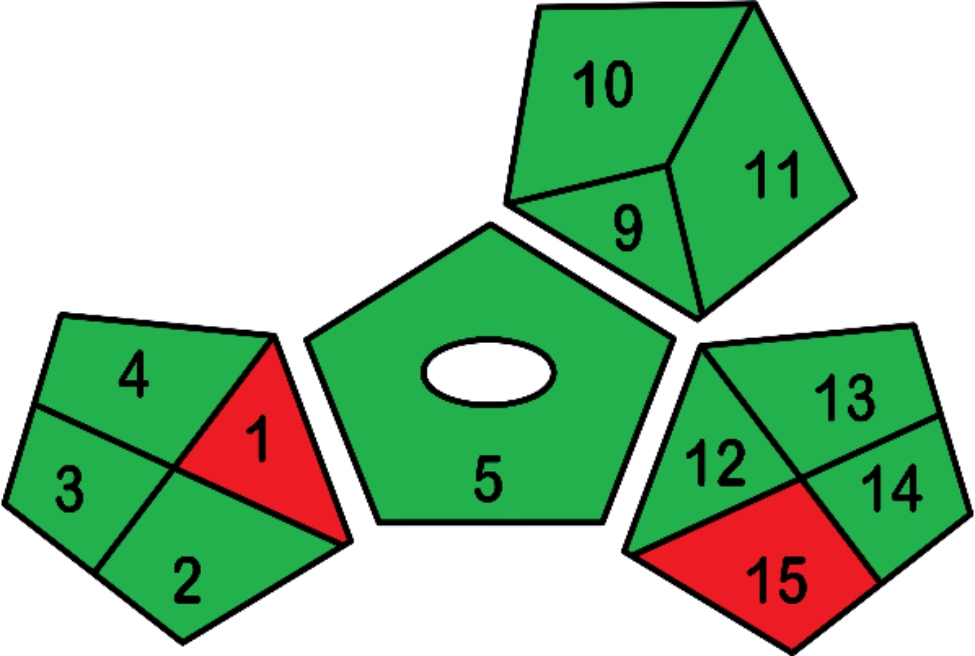
Green analytical procedure index (GAPI) tool for the proposed potentiometric sensors

GAPI is a recent developed greenness assessment method, that evaluates the greenness of entire procedure from the sample collection step till the final detection. It includes five pentagrams that represent each stage of the process. There are three color scale levels: green, yellow, or red indicating high, medium, and low environmental impact [52]. Figure 4 shows that the proposed sensors do not require sample preparation step, and capable of direct analysis resulting in removal of the sample preparation pentagram. Only two red regions were observed. On-line or in-line sample collection were not applicable. Therefore, to achieve a green method, no preservatives were used, and the samples were analyzed after collection within 24 h. There were no facilities to carry out waste treatment and this was the reason of the presence of another red region, but fortunately small amounts of waste were produced Fig. 7.

## Conclusion

Antimicrobial resistance that has been growing especially during the COVID-19 outbreak due to the excessive use of hand sanitizers and soaps. It is considered as a serious issue that the world must pay a great attention to control such devastating phenomenon. Regular monitoring of the environmental emergence of antimicrobials and the use of ecofriendly soaps and detergents is strongly recommended.

Electroanalysis is a promising green analytical technique owing to its advantages over other conventional analytical methods and can be employed for the regular monitoring of antimicrobials in the environment. Microfabrication of the used sensors with reduction of sample volume and the amount of waste produced leads to an eco-friendly method. It is worth mentioning that miniaturization of the proposed sensors offers an ease of measurement with maximum sensitivity, stability, and selectivity.

The proposed microfabricated copper ISEs allowed the ultra-trace analysis of TCS, and the incorporation of MW-CNT and β-CD proved to be successful in enhancing the sensitivity, selectivity, and stability of the proposed microfabricated sensors. Those sensors have many advantages as being cost-effective, disposable and can be embedded within lab-on a chip system during the microfabrication step for real-time continuous monitoring of TCS.

### Electronic supplementary material

Below is the link to the electronic supplementary material.

Supplementary Material 1

## Data Availability

The datasets used and/or analysed during the current study available from the corresponding author on reasonable request.
