# Supplementary material for: Eco-friendly monitoring of triclosan as an emerging antimicrobial environmental contaminant utilizing electrochemical sensors modified with CNTs nanocomposite transducer layer
Source: BMC Chem. 2023 Nov 28;17(1):170. doi: 10.1186/s13065-023-01092-0 (PMC10685535; doi:10.1186/s13065-023-01092-0)
Supplement: Supplementary file 1 — Supplementary Material 1 [file 13065_2023_1092_MOESM1_ESM.doc]

**Supplementary Material**

**for**

**“****Eco-friendly Monitoring of Triclosan as an Emerging Antimicrobial Environmental Contaminant Utilizing Electrochemical Sensors Modified with CNTs Nanocomposite Transducer Layer”**

Nardine Safwat^a^, Amr M. Mahmoud^b^*, Maha F. Abdel-Ghany^a^, Miriam F. Ayad^a^

^a^Pharmaceutical Analytical Chemistry Department, Faculty of Pharmacy, Ain Shams University, Abbassia, Cairo 11566, Egypt

^b^Pharmaceutical Analytical Chemistry Department, Faculty of Pharmacy, Cairo University, Kasr-El Aini Street, Cairo 11562, Egypt

*Corresponding Author Email: [amr.bekhet@pharma.cu.edu.eg](mailto:nardine.safwat2@pharma.asu.edu.eg)

**Literature review**

The literature has reported several chromatographic techniques for the detection of TCS in various environmental matrices; LC-MS/MS and GC-MS/MS (Hua et al. 2005; Trenholm et al. 2006; Tohidi and Cai 2015; Zang et al. 2015; Mohan and Balakrishnan 2019). Few spectrophotometric and spectrofluorimetric methods (Cabaleiro et al. 2011; Mpupa et al. 2017; Kaur et al. 2018; Montaseri and Forbes 2018) and some voltammetric methods have been reported (Yola et al. 2015; Atar et al. 2015; Regiart et al. 2016).

**Chemicals and Materials**

Sodium hydroxide (NaOH), ammonium persulphate (NH_4_S_2_O_8_), acetone, sodium chloride (NaCl), lithium bromide (LiBr), potassium chloride, (KCl), hydrochloric acid (HCl), sodium carbonate (Na_2_CO_3_), sodium bicarbonate (NaHCO_3_) and magnesium sulphate (MgSO_4_) (Prolabo, Pennsylvania, USA). Naproxen kindly supplied by National Organization of Drug Control and Research (NODCAR).

**Calibration**

Calibration curves for other interfering anions were obtained by serial dilution with deionized water. Selectivity coefficients were computed using the separate solution method (Bakker et al. 2000). Determination of the Limit of detection (LOD) was done according to the IUPAC recommendations (Lindner, E.; Umezawa 2008).

**Effect of pH**

The influence of pH on the proposed sensors’ potential response was studied at two concentration levels (10^-4^ and 10^-5^ M). Adjustment of pH over the range of (1-12) was carried out using small volumes of 1 M HCl and 1M NaOH.

The measured potential for each concentration level was plotted versus the corresponding pH.

**Effect of temperature**

The influence of temperature on the proposed sensors’ response was studied. The *emf* values of the standard working solutions in the concentration range (1 × 10^-8^ – 1 × 10^-3^ M) for sensor 1, (1 × 10^-9^ – 1 × 10^-3^ M) for sensor 2 and (1 × 10^-10^ – 1 × 10^-3^ M) for sensor 3 were recorded in the range 25 °C to 45 °C at 5 °C interval. A calibration curve was plotted at each temperature.

**Effect of foreign compounds on sensor selectivity**

The proposed sensors’ responses were evaluated in the presence of various related substances. For the evaluation of the extent to which the foreign ions could interfere with the electrodes’ response to their primary ion, the potentiometric selectivity coefficient in terms of (Log K^pot^ _primary ion, Interferent_) was utilized. The *emf* values were measured for 10^-4^ M TCS solution and 10^-4^ M interferent aqueous solution and based on the separate solution method (SSM), the selectivity coefficients could be computed using the rearranged Nicolsky-Eisenman equation (UMEZAWA et al. 2000).


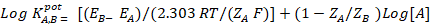


In which:

E_A_: is the potential measured in the solution of ion of interest

E_B_: is the potential measured in the solution of interferent

Z_A_, Z_B_: are the charges of the ion of interest and interferent, respectively.

(2.303 RT/ Z_A_F): is the slope of the calibration curve in mV/decade.

**Potentiometric aqueous layer test**

The long-term stability of solid state ISEs is highly affected by the formation of an aqueous layer beneath the membrane (De Marco et al. 2008). The formation of the aqueous layer can be tested by measuring the potential drift of the electrode upon changing from the ion of interest (1 × 10^-6^ M) to a highly concentrated interfering ion solution (1 × 10^-2^ M of naproxen (NPR)) and then back to the primary ion of interest. If there is an aqueous layer, a change in the membrane composition will take place and undesirable potential drifts will occur.

**Greenness evaluation**

Achievement of the analytical figures of merits including selectivity, sensitivity and lower limit of detection and green analytical procedure development has drawn the attention of the analytical community. Electroanalysis is a promising method of green analysis since it is a direct method with no need for sample collection and pretreatment steps. Miniaturization of the developed eco- friendly sensors offers the advantage of real-time measurements, the use of small sample volume and reduction of the amount of waste. The microfabrication has eliminated the use of an inner filling solution and offering the advantage of being green, simple, cheap, and sensitive. Environmental monitoring is in deep need for the development of green analytical methods instead of the conventional hazardous methods (Wang 2002; Mohamed 2015).

**Analytical eco-scale.**

It is based on assigning penalty points to various factors included in the developed method and being subtracted from a base of 100. More than 75 score will be for excellent green analysis, more than 50 for acceptable green analysis and less than 50 for inadequate green analysis. Penalty points are given to the reagent type and amount, the amount of energy of various electrical devices, the analytical waste treatment and the occupational hazard (Gałuszka et al. 2012).

**Green Analytical Procedure Index**

It is one of the recently developed greenness assessment tools. It evaluates the whole method greenness starting from the sample collection until the final determination. It consists of five pentagrams with three levels color scale for each stage including green, yellow or red ranging from high, medium and low environmental impact (Płotka-Wasylka 2018). No sample preparation is required, and direct analysis is performed resulting in removal of the sample preparation pentagram.

**References**

Atar N, Eren T, Yola ML, Wang S (2015) A sensitive molecular imprinted surface plasmon resonance nanosensor for selective determination of trace triclosan in wastewater. Sensors Actuators, B Chem 216:638–644. https://doi.org/10.1016/j.snb.2015.04.076

Baranowska I, Magiera S, Bortniczuk K (2010) Reverse-phase HPLC method for the simultaneous analysis of triclosan and triclocarban in surface waters. Water Sci Technol Water Supply 10:173–180. https://doi.org/10.2166/ws.2010.242

Cabaleiro N, Pena-Pereira F, de la Calle I, et al (2011) Determination of triclosan by cuvetteless UV-vis micro-spectrophotometry following simultaneous ultrasound assisted emulsification-microextraction with derivatization: Use of a micellar-ionic liquid as extractant. Microchem J 99:246–251. https://doi.org/10.1016/j.microc.2011.05.010

De Marco R, Veder JP, Clarke G, et al (2008) Evidence of a water layer in solid-contact polymeric ion sensors. Phys Chem Chem Phys 10:73–76. https://doi.org/10.1039/b714248j

Gałuszka A, Migaszewski ZM, Konieczka P, Namieśnik J (2012) Analytical Eco-Scale for assessing the greenness of analytical procedures. TrAC - Trends Anal Chem 37:61–72. https://doi.org/10.1016/j.trac.2012.03.013

Hua W, Bennett ER, Letcher RJ (2005) Triclosan in waste and surface waters from the upper Detroit River by liquid chromatography-electrospray-tandem quadrupole mass spectrometry. Environ Int 31:621–630. https://doi.org/10.1016/j.envint.2004.10.019

Kaur I, Gaba S, Kaur S, et al (2018) Spectrophotometric determination of triclosan based on diazotization reaction: Response surface optimization using box-behnken design. Water Sci Technol 77:2204–2212. https://doi.org/10.2166/wst.2018.123

Mohamed HM (2015) Green, environment-friendly, analytical tools give insights in pharmaceuticals and cosmetics analysis. TrAC - Trends Anal Chem 66:176–192. https://doi.org/10.1016/j.trac.2014.11.010

Mohan S, Balakrishnan P (2019) Triclosan in Treated Wastewater from a City Wastewater Treatment Plant and its Environmental Risk Assessment. Water Air Soil Pollut 230:1–13. https://doi.org/10.1007/s11270-019-4098-9

Montaseri H, Forbes PBC (2018) A triclosan turn-ON fluorescence sensor based on thiol-capped core/shell quantum dots. Spectrochim Acta - Part A Mol Biomol Spectrosc 204:370–379. https://doi.org/10.1016/j.saa.2018.06.043

Mousavi MPS, Abd El-Rahman MK, Mahmoud AM, et al (2018) In Situ Sensing of the Neurotransmitter Acetylcholine in a Dynamic Range of 1 nM to 1 mM. ACS Sensors 3:2581–2589. https://doi.org/10.1021/acssensors.8b00950

Mpupa A, Mashile GP, Nomngongo and PN (2017) Vortex assisted-supramolecular solvent microextraction coupled with spectrophotometric determination of triclosan in environmental water samples. Open Chem 15:255–262. https://doi.org/https://doi.org/10.1515/chem-2017-0032

Płotka-Wasylka J (2018) A new tool for the evaluation of the analytical procedure: Green Analytical Procedure Index. Talanta 181:204–209. https://doi.org/10.1016/j.talanta.2018.01.013

Regiart M, Magallanes JL, Barrera D, et al (2016) An ordered mesoporous carbon modified electrochemical sensor for solid-phase microextraction and determination of triclosan in environmental samples. Sensors Actuators, B Chem 232:765–772. https://doi.org/10.1016/j.snb.2016.04.031

Tohidi F, Cai Z (2015) GC/MS analysis of triclosan and its degradation by-products in wastewater and sludge samples from different treatments. Environ Sci Pollut Res 22:11387–11400. https://doi.org/10.1007/s11356-015-4289-x

Trenholm RA, Vanderford BJ, Holady JC, et al (2006) Broad range analysis of endocrine disruptors and pharmaceuticals using gas chromatography and liquid chromatography tandem mass spectrometry. Chemosphere 65:1990–1998. https://doi.org/10.1016/j.chemosphere.2006.07.004

UMEZAWA Y, BÜHLMANN P, UMEZAWA2 K, et al (2000) IUPAC Analytical chemistry division, commission on analytical nomenclature

Wang J (2002) Real-time electrochemical monitoring: Toward green analytical chemistry. Acc Chem Res 35:811–816. https://doi.org/10.1021/ar010066e

Yola ML, Atar N, Eren T, et al (2015) Sensitive and selective determination of aqueous triclosan based on gold nanoparticles on polyoxometalate/reduced graphene oxide nanohybrid. RSC Adv 5:65953–65962. https://doi.org/10.1039/c5ra07443f

Zang X, Chang Q, Hou M, et al (2015) Graphene grafted magnetic microspheres for solid phase extraction of bisphenol A and triclosan from water samples followed by gas chromatography-mass spectrometric analysis. Anal Methods 7:8793–8800. https://doi.org/10.1039/c5ay01578b
